# A Case Study Competition Among Methods for Analyzing Large Spatial Data

**DOI:** 10.1007/s13253-018-00348-w

**Published:** 2018-12-14

**Authors:** Matthew J. Heaton, Abhirup Datta, Andrew O. Finley, Reinhard Furrer, Joseph Guinness, Rajarshi Guhaniyogi, Florian Gerber, Robert B. Gramacy, Dorit Hammerling, Matthias Katzfuss, Finn Lindgren, Douglas W. Nychka, Furong Sun, Andrew Zammit-Mangion

**Affiliations:** Brigham Young University, Provo, UT USA

**Keywords:** Big data, Gaussian process, Parallel computing, Low-rank approximation

## Abstract

The Gaussian process is an indispensable tool for spatial data analysts. The onset of the “big data” era, however, has lead to the traditional Gaussian process being computationally infeasible for modern spatial data. As such, various alternatives to the full Gaussian process that are more amenable to handling big spatial data have been proposed. These modern methods often exploit low-rank structures and/or multi-core and multi-threaded computing environments to facilitate computation. This study provides, first, an introductory overview of several methods for analyzing large spatial data. Second, this study describes the results of a predictive competition among the described methods as implemented by different groups with strong expertise in the methodology. Specifically, each research group was provided with two training datasets (one simulated and one observed) along with a set of prediction locations. Each group then wrote their own implementation of their method to produce predictions at the given location and each was subsequently run on a common computing environment. The methods were then compared in terms of various predictive diagnostics. Supplementary materials regarding implementation details of the methods and code are available for this article online.

**Electronic Supplementary Material:**

Supplementary materials for this article are available at 10.1007/s13253-018-00348-w.

## Introduction

For decades, the Gaussian process (GP) has been the primary tool used for the analysis of geostatistical (point-referenced) spatial data (Schabenberger and Gotway 2004; Cressie 1993; Cressie and Wikle 2015; Banerjee et al. 2014). A spatial process $Y(\varvec{s})$ for $\varvec{s} \in \mathcal {D} \subset \mathbb {R}^2$ is said to follow a GP if any realization $\varvec{Y} = (Y(\varvec{s}_1),\dots ,Y(\varvec{s}_N))'$ at the finite number of locations $\varvec{s}_1,\dots ,\varvec{s}_N$ follows an *N*-variate Gaussian distribution. More specifically, let $\mu (\varvec{s}): \mathcal {D} \rightarrow \mathbb {R}$ denote a mean function returning the mean at location $\varvec{s}$ (typically assumed to be linear in covariates $\varvec{X}(\varvec{s}) = (1,X_1(\varvec{s}),\dots ,X_P(\varvec{s}))'$) and $\mathbb {C}(\varvec{s}_1,\varvec{s}_2): \mathcal {D}^2 \rightarrow \mathbb {R}^+$ denote a positive-definite covariance function. Then, if $Y(\varvec{s})$ follows a spatial Gaussian process, $\varvec{Y}$ has the density function,

1 $$\begin{aligned} f_{\varvec{Y}}(\varvec{y})&= \left( \frac{1}{\sqrt{2\pi }}\right) ^{N} |\varvec{\Sigma }|^{-1/2} \exp \left\{ -\frac{1}{2}(\varvec{y}-\varvec{\mu })'\varvec{\Sigma }^{-1}(\varvec{y}-\varvec{\mu })\right\} \end{aligned}$$

where $\varvec{\mu } = (\mu (\varvec{s}_1),\dots ,\mu (\varvec{s}_N))'$ is the mean vector and $\varvec{\Sigma } = \{\mathbb {C}(\varvec{s}_i,\varvec{s}_j)\}_{ij}$ is the $N\times N$ covariance matrix governed by $\mathbb {C}(\varvec{s}_i,\varvec{s}_j)$ (e.g., the Matérn covariance function). From this definition, the appealing properties of the Gaussian distribution (e.g., Gaussian marginal and conditional distributions) have rendered the GP an indispensable tool for any spatial data analyst to perform such tasks as kriging (spatial prediction) and proper uncertainty quantification.

With the modern onset of larger and larger spatial datasets, however, the use of Gaussian processes for scientific discovery has been hindered by computational intractability. Specifically, evaluating the density in (1) requires $\mathcal {O}(N^3)$ operations and $\mathcal {O}(N^2)$ memory which can quickly overwhelm computing systems when *N* is only moderately large. Early solutions to this problem included factoring (1) into a series of conditional distributions (Vecchia 1988; Stein et al. 2004), the use of pseudo-likelihoods (Varin et al. 2011; Eidsvik et al. 2014), modeling in the spectral domain (Fuentes 2007) or using tapered covariance functions (Furrer et al. 2006; Kaufman et al. 2008; Stein 2013). Beginning in the late 2000’s, several approaches based on low-rank approximations to Gaussian processes were developed (or became popular) including discrete process convolutions (Higdon 2002; Lemos and Sansó 2009), fixed rank kriging (Cressie and Johannesson 2008; Kang and Cressie 2011; Katzfuss and Cressie 2011), predictive processes (Banerjee et al. 2008; Finley et al. 2009), lattice kriging (Nychka et al. 2015) and stochastic partial differential equations (Lindgren et al. 2011). Sun et al. (2012), Bradley et al. (2016) and Liu et al. (2018) provide exceptional reviews of these methods and demonstrate their effectiveness for modeling spatial data.

After several years of their use, however, scientists have started to observe shortcomings in many of the above methods for approximating GPs such as the propensity to oversmooth the data (Simpson et al. 2012; Stein 2014) and even, for some of these methods, an upper limit on the size of the dataset that can be modeled. Hence, recent scientific research in this area has focused on the efficient use of modern computing platforms and the development of methods that are parallelizable. For example, Paciorek et al. (2015) show how (1) can be calculated using parallel computing while Katzfuss and Hammerling (2017) and Katzfuss (2017) develop a basis-function approach that lends itself to distributed computing. Alternatively, Barbian and Assunção (2017) and Guhaniyogi and Banerjee (2018) propose dividing the data into a large number of subsets, draw inference on the subsets in parallel and then combining the inferences. Datta et al. (2016a, c) build upon Vecchia (1988) by developing novel approaches to factoring (1) as a series of conditional distributions based only on nearest neighbors.

Given the plethora of choices to analyze large spatially correlated data, for this paper, we seek to not only provide an overview of modern methods to analyze massive spatial datasets, but also lightly compare the methods in a unique way. Specifically, this research implements the common task framework of Wikle et al. (2017) by describing the outcome of a friendly case study competition between various research groups across the globe who each implemented their own method to analyze the same spatial datasets (see the list of participating groups in Table 1). That is, several research groups were provided with two spatial datasets (one simulated and one real) with a portion of each dataset removed to validate predictions (research groups were not provided with the removed portion so that this study is “blinded”). The simulated data represent a scenario where the Gaussian process assumption is valid (i.e., a correctly specified model), whereas the real dataset is a scenario when the model is potentially mis-specified due to inherent non-stationarity or non-Gaussian errors. Each group then implemented their unique method and provided a prediction (and prediction interval or standard error) of the spatial process at the held out locations. The predictions were compared by a third party and are summarized herein.

The case study competition described herein is unique and novel in that, typically, comparisons/reviews of various methods is done by a single research group implementing each method (see Sun et al. 2012; Bradley et al. 2016). However, single research groups may be more or less acquainted with some methods leading to a possibly unfair comparison with those methods they are less familiar with. In contrast, for the comparison/competition here, each method was implemented by a research group with strong expertise in the method and who is well-versed in any possible intricacies associated with its use. Further, unlike the previous reviews of Sun et al. (2012); Bradley et al. (2016), we provide a comparison of each method’s ability to quantify the uncertainty associated with predictions. Hence, in terms of scientific contributions, this paper (i) serves as a valuable review, (ii) discusses a unique case study comparison of spatial methods for large datasets, (iii) provides code to implement each method to practitioners (see supplementary materials), (iv) provides a comparison of the uncertainty quantification associated with each method and (v) establishes a framework for future studies to follow when comparing various analytical methods.

The remainder of this paper is organized as follows. Section 2 gives a brief background on each method. Section 3 provides the setting for the comparison along with background on the datasets. Section 4 then summarizes the results of the comparison in terms of predictive accuracy, uncertainty quantification and computation time. Section 5 draws conclusions from this study and highlights future research areas for the analysis of massive spatial data.

## Overview of Methods for Analyzing Large Spatial Data

This section contains a brief overview of the competitors in this case study competition. For convenience, we group the methods into one of the following categories: (i) low rank, (ii) sparse covariance matrices, (iii) sparse precision matrices and (iv) algorithmic. The low-rank approaches are so classified because these typically involve reducing the rank of the $N\times N$ matrix ${\varvec{\Sigma }}$. Sparse covariance methods work by introducing “0’s” into ${\varvec{\Sigma }}$ allowing for sparse matrix computations. Sparse precision methods, in contrast, induce sparsity in the precision matrix to allow for efficient computation. Algorithmic approaches (which is perhaps the most vaguely defined category) differ from the previous approaches in that they focus more on a transductive approach to learning by focusing more on fitting schemes than model building. Importantly, we introduce these categories as a subjective classification purely for clarity in exposition. As with any subjective grouping, a single method may include pieces of various categories. As such, we strongly encourage viewing the method as a whole rather than solely through the lens of our subjective categorization.

### Low-Rank Methods

#### Fixed Rank Kriging

Fixed Rank Kriging (FRK, Cressie and Johannesson 2006, 2008) is built around the concept of a spatial random effects (SRE) model. In FRK, the process $\widetilde{Y}(\varvec{s}), \varvec{s} \in \mathcal {D},$ is modeled as

2 $$\begin{aligned} \widetilde{Y}(\varvec{s}) = \mu (\varvec{s}) + w(\varvec{s}) + \xi (\varvec{s}),\quad \varvec{s} \in \mathcal {D}, \end{aligned}$$

where $\mu (\varvec{s})$ is the mean function that is itself modeled as a linear combination of known covariates (i.e., $\mu (\varvec{s}) = \varvec{X}'(\varvec{s})\varvec{\beta }$ where $\varvec{X}(\varvec{s})$ is a vector of covariates evaluated at location $\varvec{s}$ and $\varvec{\beta }$ are the associated coefficients), $w(\varvec{s})$ is a smooth process, and $\xi (\varvec{s})$ is a fine-scale process, modeled to be approximately spatially uncorrelated with variance $\sigma ^2_\xi v(\varvec{s})$ where $v(\varvec{s})$ is a known weighting function. The process $\xi (\varvec{s})$ in (2) is designed to soak up variability in $\widetilde{Y}(\varvec{s})$ not accounted for by $w(\varvec{s})$.

The primary assumption of FRK is that the spatial process $w(\cdot )$ can be approximated by a linear combination of *K* basis functions $\varvec{h}(\varvec{s}) = (h_1(\varvec{s}),\dots ,h_K(\varvec{s}))', \varvec{s} \in \mathcal {D},$ and *K* basis-function coefficients $\varvec{w}^\star = (w_1^\star ,\dots ,w_K^\star )'$ such that,

3 $$\begin{aligned} w(\varvec{s}) \approx {\widetilde{w}}(\varvec{s}) = \sum _{k=1}^K h_k(\varvec{s})w^\star _k, \quad \varvec{s} \in \mathcal {D}. \end{aligned}$$

The use of *K* basis functions ensures that all estimation and prediction equations only contain inverses of matrices of size $K \times K$, where $K \ll N$. In practice, the set $\{h_k(\varvec{\cdot })\}$ in (3) is comprised of functions at *R* different resolutions such that (3) can also be written as

4 $$\begin{aligned} {\widetilde{w}}(\varvec{s}) = \sum _{r=1}^R\sum _{k=1}^{K_r} h_{rk}(\varvec{s})w_{rk}^\star ,\quad \varvec{s} \in \mathcal {D}, \end{aligned}$$

where $h_{rk}(\varvec{s})$ is the $k\mathrm{th}$ spatial basis function at the $r\mathrm{th}$ resolution with associated coefficient $w^\star _{rk}$, and $K_r$ is the number of basis functions at the $r\mathrm{th}$ resolution, such that $K=\sum _{r=1}^R K_r$ is the total number of basis functions used. In the experiments we used $R=3$ resolutions of bisquare basis functions and a total of $K = 475$ basis functions.

The coefficients $\varvec{w}^\star = (w^\star _{rk}: r = 1,\dots ,R;~k = 1,\dots , K_r)'$ have as covariance matrix $\mathbb {V}\text {ar}(\varvec{w}^\star ) = \varvec{\Sigma }_{w^\star }(\varvec{\theta })$, where $\varvec{\theta }$ are parameters that need to be estimated. In this work, $\varvec{\Sigma }_{w^\star }(\varvec{\theta })$ is a block-diagonal matrix composed from *R* dense matrices, where the $r\mathrm{th}$ block has $(i,j)\mathrm{th}$ element $\sigma ^2_r\exp (-d_r(i,j)/\phi _r)$ and where $d_r(i,j)$ is the distance between the centroids of the $i\mathrm{th}$ and $j\mathrm{th}$ basis function at the $r\mathrm{th}$ resolution; $\sigma ^2_r$ is the variance at the $r\mathrm{th}$ resolution; $\phi _r$ is the spatial correlation parameter of the exponential correlation function at the $r\mathrm{th}$ resolution; and $\varvec{\theta } = (\sigma ^2_1,\dots ,\sigma ^2_R,\phi _1,\dots ,\phi _R)'$. Note that $\varvec{\Sigma }_{w^\star }(\varvec{\theta })$ can also be unstructured in which case $K(K+1)/2$ parameters need to be estimated; however, this case is not considered here.

There are several variants of FRK. In this work, we use the implementation by Zammit-Mangion and Cressie (2018) which comes in the form of the R package FRK, available from the Comprehensive R Archive Network (CRAN). In this paper we utilize v0.1.6 of that package. In FRK, the process evaluated at $\varvec{s}_i$, $\widetilde{Y}(\varvec{s}_i)$, is assumed to be observed with measurement error $\varepsilon (\varvec{s}_i)$. The data model is therefore given by

5 $$\begin{aligned} Y(\varvec{s}_i) = \mu (\varvec{s}_i) + {\widetilde{w}}(\varvec{s}_i) + \xi (\varvec{s}_i) + \varepsilon (\varvec{s}_i), \quad i = 1,\dots , N, \end{aligned}$$

where $\varepsilon (\varvec{s}_i)$ denotes independent and identically normally distributed measurement error with mean 0 and measurement-error variance $\sigma ^2_\varepsilon$. Under this specification, the joint model for $Y(\cdot )$ evaluated at all *N* observed locations is,

6 $$\begin{aligned} \varvec{Y} = {\varvec{X}}\varvec{\beta }+{\varvec{H}}\varvec{w}^\star + \varvec{\xi } + \varvec{\varepsilon }, \end{aligned}$$

where ${\varvec{X}}$ is the design matrix; $\varvec{\beta }$ are the regression coefficients; ${\varvec{H}}$ is the $N\times K$ matrix of spatial basis functions with associated random coefficients $\varvec{w}^\star \sim \mathcal {N}(\varvec{0},\varvec{\Sigma }_{w^\star }(\varvec{\theta }))$; $\varvec{\xi } \sim \mathcal {N}(\varvec{0},\sigma ^2_\xi {\varvec{D}})$ with ${\varvec{D}}$ being a known, diagonal weight matrix specified by the user (here we just use ${\varvec{D}} = {\varvec{I}}$ but this need not be the case); and $\varvec{\varepsilon } \sim \mathcal {N}(\varvec{0},\sigma ^2_\varepsilon {\varvec{i}})$. The package FRK is used to first estimate $\varvec{\theta }, \sigma ^2_\xi$ and $\sigma ^2_\varepsilon$ using a combination of semivariogram and maximum-likelihood techniques (see Kang et al. 2009) and, subsequently, do prediction with the estimated parameters ‘plugged-in.’ More details on the implementation of FRK for this study are included in the supplementary materials.

#### Predictive Processes

For the predictive-process (PP) approach, let $\varvec{s}^\star _1,\dots ,\varvec{s}^\star _K$ denote a set of “knot” locations well dispersed over the spatial domain $\mathcal {D}$. Assume that the SREs ($w(\varvec{s})$) in (2) follow a mean zero Gaussian process with covariance function $\mathbb {C}(\varvec{s},\varvec{s}') = \sigma ^2_w \rho (\varvec{s},\varvec{s}')$ where $\rho (\cdot ,\cdot )$ is a positive-definite correlation function. Under this Gaussian process assumption, the SREs $\varvec{w}^\star = (w(\varvec{s}^\star _1),\dots ,w(\varvec{s}^\star _K))' \sim \mathcal {N}(0,{\varvec{\Sigma }}_{w^\star })$ where ${\varvec{\Sigma }}_{w^\star }$ is a $K\times K$ covariance matrix with $ij\mathrm{th}$ element $\mathbb {C}(\varvec{s}^\star _i,\varvec{s}_j^\star )$. The PP approach exploits the Gaussian process assumption for the SREs and replaces $w(\varvec{s})$ in (2) with

7 $$\begin{aligned} {\widetilde{w}}(\varvec{s}) = \mathbb {C}'(\varvec{s},\varvec{s}^\star ){\varvec{\Sigma }}_{w^\star }^{-1}\varvec{w}^\star \end{aligned}$$

where $\mathbb {C}(\varvec{s},\varvec{s}^\star ) = (\mathbb {C}(\varvec{s},\varvec{s}_1^\star ),\dots ,\mathbb {C}(\varvec{s},\varvec{s}_K^\star ))'$. Note that (7) can be equivalently written as the basis function expression given above in (3) where the basis functions are $\mathbb {C}(\varvec{s},\varvec{s}^\star ){\varvec{\Sigma }}_{w^\star }^{-1}$ and $\varvec{w}^\star$ effectively plays the role of the basis coefficients.

In the subsequent analyses presented in Sect. 4, we applied a fairly coarse 14$\times$14 knot grid in an attempt to balance computing time with predictive performance. Increasing the number of knots beyond 196 will improve inference, at the cost of longer run time.

Finley et al. (2009) noted that the basis-function expansion in (7) systematically underestimates the marginal variance $$\sigma ^2_w$$ from the original process. That is, $$\mathbb {V}\text {ar}({\widetilde{w}}(\varvec{s})) = \mathbb {C}'(\varvec{s},\varvec{s}^\star ){\varvec{\Sigma }}_{w^\star }^{-1}\mathbb {C}'(\varvec{s},\varvec{s}^\star ) $$$$\le \sigma ^2_w$$. To counterbalance this underestimation of the variance, Finley et al. (2009) use the structure in (5),

8 $$\begin{aligned} Y(\varvec{s}) = \mu (\varvec{s}) + {\widetilde{w}}(\varvec{s}) + \xi (\varvec{s}) + \varepsilon (\varvec{s}) \end{aligned}$$

where $\xi (\varvec{s})$ are spatially independent with distribution $\mathcal {N}(0,\sigma ^2_w-\mathbb {C}'(\varvec{s},\varvec{s}^\star ){\varvec{\Sigma }}_{w^\star }^{-1}\mathbb {C}'(\varvec{s},\varvec{s}^\star ))$ such that $\mathbb {V}\text {ar}({\widetilde{w}}(\varvec{s}) + \xi (\varvec{s})) = \sigma ^2_w$ as in the original parent process. This adjustment in (8) is called the “modified” predictive process and is what is used in this competition.

As with FRK, the associated likelihood under (8) only requires calculating the inverse and determinant of a dense $K\times K$ matrix and diagonal $N\times N$ matrices which results in massive computational savings when $K \ll N$. However, one advertised advantage of using the PP approach as opposed to FRK or LatticeKrig is that the PP basis functions are completely determined by the choice of covariance function $\mathbb {C}(\cdot ,\cdot )$. Hence, the PP approach is unaltered even when considering modeling complexities such as anisotropy, non-stationarity or even multivariate processes. At the same time, however, when $\mathbb {C}(\cdot ,\cdot )$ is governed by unknown parameters (which is nearly always the case) the PP basis functions need to be calculated iteratively rather than once as in FRK or LatticeKrig which will subsequently increase computation time.

### Sparse Covariance Methods

#### Spatial Partitioning

Let the spatial domain $\mathcal {D} = \bigcup _{d=1}^D \mathcal {D}_d$ where $\mathcal {D}_1,\dots ,\mathcal {D}_D$ are subregions that form a partition (i.e., $\mathcal {D}_{d_1} \bigcap \mathcal {D}_{d_2} = \emptyset$ for all $d_1 \ne d_2$). The modeling approach based on spatial partitioning is to again assume the model in (6) but take on the assumption of independence between observations across subregions. More specifically, if $\varvec{Y}_d = \{Y(\varvec{s}_i): \varvec{s}_i \in \mathcal {D}_d\}$ where $d=1,\dots ,D$, then

9 $$\begin{aligned} \varvec{Y}_d&= {\varvec{X}}_d\varvec{\beta }+{\varvec{H}}_d\varvec{w}^\star +\varvec{\xi }_d + \varvec{\varepsilon }_d \end{aligned}$$

where ${\varvec{X}}_d$ is a design matrix containing covariates associated with $\varvec{Y}_d$, ${\varvec{H}}_d$ is a matrix of spatial basis functions (such as those used in predictive processes, fixed rank kriging or lattice kriging mentioned above) and $\varvec{\xi }_d$ and $\varvec{\varepsilon }_d$ are the subvectors of $\varvec{\xi }$ and $\varvec{\varepsilon }$ corresponding to region *d*. Notice that, in (9) each subregion shares common $\varvec{\beta }$ and $\varvec{w}^\star$ parameters which allows smoothing across subregions in spite of the independence assumption. Further, the assumption of independence across subregions effectively creates a block-diagonal structure for ${\varvec{\Sigma }}$ and allows the likelihood to be computed in parallel (with one node per subregion) thereby facilitating computation.

By way of distinction, this approach is inherently different from the “divide and conquer” approach (Liang et al. 2013; Barbian and Assunção 2017). In the divide and conquer approach, the full dataset is subsampled, the model is fit to each subset and the results across subsamples are pooled. In contrast, the spatial partition approach uses all the data simultaneously in obtaining estimates, but the independence across regions facilitates computation.

The key to implementing the spatial partitioning approach is the choice of partition, and the literature is replete with various options. A priori methods to define the spatial partitioning include partitioning the region into equal areas (Sang et al. 2011), partitioning based on centroid clustering (Knorr-Held and Raßer 2000; Kim et al. 2005) and hierarchical clustering based on spatial gradients (Anderson et al. 2014; Heaton et al. 2017). Alternatively, model-based approaches to spatial partitioning include treed regression (Konomi et al. 2014) and mixture modeling (Neelon et al. 2014), but these approaches typically require more computation. For this analysis, a couple of different partitioning schemes were considered, but each scheme resulted in approximately equivalent model fit to the training data. Hence, based on the results from the training data, for the competition below we used an equal area partition of approximately 6000 observations per subregion.

#### Covariance Tapering

The idea of covariance tapering is based on the fact that many entries in the covariance matrix $\varvec{\Sigma }$ in (1) are close to zero and associated location pairs could be considered as essentially independent. Covariance tapering multiplies the covariance function $\mathbb {C}(\varvec{s}_i,\varvec{s}_j)$ with a compactly supported covariance function, resulting in another positive-definite covariance function but with compact support. From a theoretical perspective, covariance tapering (in the framework of infill-asymptotics) is using the concept of Gaussian equivalent measures and mis-specified covariance functions (see, e.g., Stein 1999 and references therein). Subsequently, Furrer et al. (2006) have assumed a second-order stationary and isotropic Matérn covariance to show asymptotic optimality for prediction under tapering. This idea has been extended to different covariance structures (Stein 2013), non-Gaussian response (Hirano and Yajima 2013) and multivariate and/or spatiotemporal setting (Furrer et al. 2016).

From a computational aspect, the compact support of the resulting covariance function provides the computational savings needed by employing sparse matrix algorithms to efficiently solve systems of linear equations. More precisely, to evaluate density (1), a Cholesky factorization for ${\varvec{\Sigma }}$ is performed followed by two solves of triangular systems. For typical spatial data settings, the solve algorithm is effectively linear in the number of observations.

For parameter estimation in the likelihood framework, one- and two-taper approaches exist (see Kaufman et al. 2008; Du et al. 2009; Wang and Loh 2011; Bevilacqua et al. 2016, for relevant literature). To distinguish the two approaches, notice that the likelihood in (1) can be rewritten as

10 $$\begin{aligned} f_{\varvec{Y}}(\varvec{y})&= \left( \frac{1}{\sqrt{2\pi }}\right) ^{N} |\varvec{\Sigma }|^{-1/2} \text {etr}\left\{ -\frac{1}{2}(\varvec{y}-\varvec{\mu })(\varvec{y}-\varvec{\mu })'{\varvec{\Sigma }}^{-1}\right\} \end{aligned}$$

where $\text {etr}({\varvec{A}}) = \exp (\text {trace}(A))$. In the one-taper setting, only the covariance is tapered such that ${\varvec{\Sigma }}$ in (10) is replaced by ${\varvec{\Sigma }}\odot {\varvec{T}}$ where “$\odot$” denotes the Hadamard product and ${\varvec{T}}$ is the $N\times N$ tapering matrix. In the two-tapered approach both the covariance and empirical covariance are affected such that not only is ${\varvec{\Sigma }}$ replaced by ${\varvec{\Sigma }}\odot {\varvec{T}}$ but $(\varvec{y}-\varvec{\mu })(\varvec{y}-\varvec{\mu })'$ is replaced by $(\varvec{y}-\varvec{\mu })(\varvec{y}-\varvec{\mu })' \odot {\varvec{T}}$. The one-taper equation results in biased estimates of model parameters while the two-taper approach is based on estimating equations (and is, therefore, unbiased) but comes at the price of a severe loss of computational efficiency. If the one-taper biased estimates of model parameters are used for prediction, the biases may result in some loss of predictive accuracy (Furrer et al. 2016).

Although tapering can be adapted to better take into account uneven densities of locations and complex anisotropies, we use a simple straightforward approach for this competition. The implementation here relies almost exclusively on the R package spam (Furrer and Sain 2010; Furrer 2016). Alternatively to likelihood approaches and in view of computational costs, we have minimized the squared difference between an empirical covariance and parameterized covariance function. The gridded structure of the data is exploited and the empirical covariance is estimated for a specific set of locations only; and thus is close to classical variogram estimation and fitting (Cressie 1993).

### Sparse Precision Methods

#### LatticeKrig

LatticeKrig (LK, Nychka et al. 2015) uses nearly the same setup as is employed by FRK. Specifically, LK assumes the model (6) but omits the fine-scale process $\xi (\cdot )$. LatticeKrig also follows the multiresolution approach in (4) for the matrix ${\varvec{H}}$, but LK uses a different structure and constraints than FRK. First, the marginal variance of each resolution $\varvec{h}_{r}'(\varvec{s})\varvec{w}_r^\star$ where $\varvec{h}_r'(\varvec{s}) = (h_{r1}(\varvec{s}),\dots ,h_{rK_r}(\varvec{s}))'$ are the basis functions of the $r\mathrm{th}$ resolution with coefficients $\varvec{w}^\star _{r} = (w^\star _{r1},\dots ,w^\star _{rK_r})'$ is constrained to be $\sigma ^2_{w^\star }\alpha _r$ where $\sigma ^2_{w^\star },\alpha _r>0$ and $\sum _{r=1}^R\alpha _r = 1$. To further reduce the number of parameters, LK sets $\alpha _r \sim r^{-\nu }$ where $\nu$ is a single free parameter.

LatticeKrig obtains multiresolution radial basis functions by translating and scaling a radial function in the following manner. Let $\varvec{u}_{rk}$ for $r=1,\dots ,R$ and $k=1,\dots ,K_r$ denote a regular grid of $K_r$ points on $\mathcal {D}$ corresponding to resolution *r*. For this article, LK defines

11 $$\begin{aligned} h_{rk}(\varvec{s}) = \psi (\Vert \varvec{s}-\varvec{u}_{rk}\Vert /\lambda _r) \end{aligned}$$

where the distance is taken to be Euclidean because the spatial region in this case is of small geographic extent and $\lambda _r = 2^{-r}$. Further, LK defines

12 $$\begin{aligned} \psi (d) \propto {\left\{ \begin{array}{ll}\frac{1}{3}(1-d)^6 ( 35d^2 + 18d+3) &{} \text { if } d \le 1\\ 0 &{} \text { otherwise.} \end{array}\right. } \end{aligned}$$

which are Wendland polynomials and are positive definite (an attractive property when the basis is used for interpolation). Finally, the basis functions in (12) are normalized at each resolution so that the process marginal variance at all $\varvec{s}$ is $\sigma ^2_{w^\star } \alpha _r$. This reduces edge effects and makes for a better approximation to a stationary covariance function.

LatticeKrig assumes the coefficients at each resolution $\varvec{w}^\star _{r} = (w^\star _{r1},\dots ,w^\star _{rK_r})'$ are independent (similar to the block-diagonal structure used in FRK) and follow a multivariate normal distribution with covariance $\varvec{Q}_r^{-1}(\phi _r)$ parameterized by a single parameter $\phi _r$. Because the locations $\{\varvec{u}_{rk}\}_{k=1}^{K_r}$ are prescribed to be a regular grid, LK uses a spatial autoregression/Markov random field (see Banerjee et al. 2014, Section 4.4) structure for $\varvec{Q}_r^{-1}(\phi _r)$ leading to sparsity and computational tractability. Furthermore, because $\varvec{Q}_r(\phi _r)$ is sparse, LK can set *K* to be very large (as in this competition where $K=136,000 > N$) without much additional computational cost. The supplementary material to this article contains additional information about the implementation of LatticeKrig used in this case study.

#### Multiresolution Approximations

The multiresolution approximation (MRA) can be viewed as a combination of several previously described approaches. Similar to FRK or LatticeKrig, the MRA also uses the basis-function approach in (4) but uses *compactly* supported basis functions at different resolutions. In contrast to FRK or LatticeKrig, the MRA basis functions and the prior distribution of the corresponding weights are chosen using the predictive-process approach to automatically adapt to any given covariance function $\mathbb {C}(\cdot )$, and so the MRA can adjust flexibly to a desired spatial smoothness and dependence structure. Scalability of the MRA is ensured in that for increasing resolution, the number of basis functions increases while the support of each function (i.e., the part of the spatial domain in which it is nonzero) decreases allowing the number of basis functions to be approximately the same as the data. Decreasing support (and increasing sparsity of the covariance matrices of the corresponding weights) is achieved either by increasingly severe tapering of the covariance function (MRA-taper; Katzfuss and Gong 2017) or by recursively partitioning the spatial domain (MRA-block; Katzfuss 2017). This can lead to (nearly) exact approximations with quasilinear computational complexity.

While the MRA-taper has some attractive smoothness properties, we focus here on the MRA-block which is based on a recursive partitioning of the domain $\mathcal {D}$ into smaller and smaller subregions up to some level *M*. Within each (sub-)region at each resolution, there is a small number, say $r_0$, of basis functions. The resulting approximation of the process (including its variance and smoothness) in each region at resolution *M* is exact. In addition, it is feasible to compute and store the joint posterior covariance matrix (i.e., not just its inverse as with related approaches) for a large number of prediction locations as a product of two sparse matrices (Jurek and Katzfuss 2018).

The MRA-block is designed to take full advantage of high-performance computing systems, in that inference is well suited for massively distributed computing, with limited communication overhead. The computational task is split into small parts by assigning a computational node to each region of the recursive partitioning. The nodes then deal in parallel with the basis functions corresponding to their assigned regions leading to a polylogarithmic computational complexity. For this project, we use $M=9$ levels, partition each domain in 2 parts and set the number of basis function in each partition to $r_0=64$.

#### Stochastic PDEs

The stochastic partial differential equation approach (SPDE) is based on the equivalence between Matérn covariance fields and stochastic PDEs, in combination with the Markov property that on two-dimensional domains holds for integer valued smoothness parameters in the Matérn family. The starting point is a basis expansion for $w(\varvec{s})$ of the form (3), where the basis functions $h_k(\varvec{s})$ are chosen to be piecewise linear on a triangulation of the domain (Lindgren et al. 2011). The optimal joint distribution for the $w_k^\star$ coefficients is obtained through a finite element construction, which leads to a sparse inverse covariance matrix (precision) $\varvec{Q}_\theta (\varvec{\phi })$. The precision matrix elements are polynomials in the precision and inverse range parameters ($1/\phi _{\sigma }^2$ and $1/\phi _r$), with sparse matrix coefficients that are determined solely by the choice of triangulation. This differs from the sequential Markov construction of the NNGP method which instead constructs a square-root-free $\varvec{L}\varvec{D}\varvec{L}'$ Cholesky decomposition of its resulting precision matrix (in a reverse order permutation of the elements).

The spatial process is specified through a joint Gaussian model for $\varvec{z}=(\varvec{w}^\star ,\, \varvec{\beta })$ with prior mean $\varvec{0}$ and block-diagonal precision $\varvec{Q}_z=\text {diag}(\varvec{Q}_{w^\star },\varvec{Q}_\beta )$, where $\varvec{Q}_\beta =\varvec{I}\cdot 10^{-8}$ gives a vague prior for $\varvec{\beta }$. Introducing the sparse basis evaluation matrix $\varvec{H}$ with elements $H_{ij}=h_j(\varvec{s}_i)$ and covariate matrix $\varvec{X}=X_j(\varvec{s}_i)$, the observation model is then $\varvec{Y} = \varvec{X}\varvec{\beta } + \varvec{H} \varvec{w}^\star + \varvec{\varepsilon } = {\varvec{A}}\varvec{z} + \varvec{\varepsilon }$ where $\varvec{A}=(\varvec{H},\,\varvec{X})$, and $\varvec{\varepsilon }$ is a zero mean observation noise vector with diagonal precision $\varvec{Q}_\varepsilon =\varvec{I}/\sigma _\varepsilon ^2$.

Using the precision based equations for multivariate Normal distributions, the conditional precision and expectation for $\varvec{z}$ are given by $\varvec{Q}_{z|y} = \varvec{Q}_z + \varvec{A'} \varvec{Q}_\varepsilon \varvec{A}$ and $\varvec{\mu }_{z|y} = \varvec{Q}_{z|y}^{-1} \varvec{A'} \varvec{Q}_\varepsilon \varvec{Y}$, where sparse Cholesky factorisation of $\varvec{Q}_{z|y}$ is used for the linear solve. The elements of $\varvec{z}$ are automatically reordered to keep the Cholesky factors as sparse as possible. The resulting computational and storage cost for the posterior predictions and multivariate Gaussian likelihood of a spatial Gaussian Markov random field of this type with *K* basis functions is $\mathcal {O}(K^{3/2})$. Since the direct solver does not take advantage of the stationarity of the model, the same prediction cost would apply to non-stationary models. For larger problems, more easily parallelizable iterative sparse solvers (e.g., multigrid) can be applied, but for the relatively small size of the problem here, the straightforward implementation of a direct solver is likely preferable.

The implementation of the SPDE method used here is based on the R package INLA (Rue et al. 2017), which is aimed at Bayesian inference for latent Gaussian models (in particular Bayesian generalized linear, additive, and mixed models) using integrated nested Laplace approximations (Rue et al. 2009). The parameter optimization for $\varvec{\phi }=(\phi _{r},\phi _{\sigma },\sigma _\varepsilon ^2)$ uses general numerical log-likelihood derivatives, thus the full Bayesian inference was therefore turned off, leading to an empirical Bayes estimate of the covariance parameters. Most of the running time is still spent on parameter optimization, but using the same parameter estimation technique as for LK, in combination with a purely Gaussian implementation, substantively reduces the total running time even without specialized code for the derivatives.

#### Nearest Neighbor Processes

The nearest neighbor Gaussian process (NNGP) developed in Datta et al. (2016a, b) is defined from the conditional specification of the joint distribution of the SREs in (2). Let $w(\varvec{s})$ in (2) follow a mean zero Gaussian process with $\mathbb {C}(\varvec{s},\varvec{s}') = \sigma ^2_w\rho (\varvec{s},\varvec{s}')$ where $\rho (\cdot )$ is a positive-definite correlation function. Factoring the joint distribution of $w(\varvec{s}_1),\dots ,w(\varvec{s}_N)$ into a series of conditional distributions yields that $w(\varvec{s}_1) = 0+\eta (\varvec{s}_1)$ and

13 $$\begin{aligned} w(\varvec{s}_i) \mid \varvec{w}_{1:(i-1)} = \mathbb {C}'(\varvec{s}_1,\varvec{s}_{1:(i-1)}){\varvec{\Sigma }}_{1:(i-1)}^{-1} \varvec{w}_{1:(i-1)} + \eta (\varvec{s}_i) \end{aligned}$$

where $\varvec{w}_{1:(i-1)} = (w(\varvec{s}_1),\dots ,w(\varvec{s}_{i-1}))'$, $\mathbb {C}(\varvec{s}_1,\varvec{s}_{1:(i-1)}) = (\mathbb {C}(\varvec{s}_i,\varvec{s}_1),\dots ,\mathbb {C}(\varvec{s}_i,\varvec{s}_{i-1})'$, ${\varvec{\Sigma }}_{1:(i-1)} = \mathbb {V}\text {ar}(\varvec{w}_{1:(i-1)})$ and $\eta$’s are independent, mean zero, normally distributed random variables. More compactly, (13) is equivalent to $\varvec{w} = \varvec{A} \varvec{w} + \varvec{\eta }$ where $\varvec{A} = (a_{ij})$ is a lower triangular matrix with zeroes along the diagonal and $\varvec{\eta }= (\eta (\varvec{s}_1),\dots ,\eta (\varvec{s}_n))' \sim N(0, \varvec{D})$ with diagonal entries $\mathbb {C}(\varvec{s}_i,\varvec{s}_i) - \mathbb {C}'(\varvec{s}_1,\varvec{s}_{1:(i-1)}){\varvec{\Sigma }}_{1:(i-1)}^{-1}\mathbb {C}(\varvec{s}_1,\varvec{s}_{1:(i-1)})$. This effectuates a joint distribution $\varvec{w} \sim N(0, \varvec{\Sigma })$ where $\varvec{\Sigma }^{-1} = (\varvec{I} - \varvec{A})'\varvec{D} ^ {-1} (\varvec{I} - \varvec{A})$. Furthermore, when predicting for any $\varvec{s} \notin \{\varvec{s}_1,\dots ,\varvec{s}_N\}$, one can define

14 $$\begin{aligned} w(\varvec{s})\mid \varvec{w}_{1:N} = \varvec{a}'(s)\varvec{w}_{1:N} + \eta (\varvec{s}) \end{aligned}$$

similar to (13).

A sparse formulation of $\varvec{A}$ ensures that evaluating the likelihood of $\varvec{w}$ (and, hence, of $\varvec{Y}$) will be computationally scalable because ${\varvec{\Sigma }}^{-1}$ is sparse. Because spatial covariances decrease with increasing distance, Vecchia (1988) demonstrated that replacing the conditional set $\varvec{w}_{1:(i-1)}$ by the smaller set of *m* nearest neighbors (in terms of Euclidean distance) of $\varvec{s}_i$ provides an excellent approximation to the conditional density in (13). Datta et al. (2016a) demonstrated that this is equivalent to $\varvec{A}$ having at-most *m* nonzero entries in each row (in this study we take $m=25$) and thereby corresponds to a proper probability distribution. Similarly, for prediction at a new location $\varvec{s}$, a sparse $\varvec{a}(\varvec{s})$ in (14) is constructed based on *m*-nearest neighbors of $\varvec{s}$ among $\varvec{s}_1, \dots , \varvec{s}_N$. The resulting Gaussian process is referred to as the Nearest Neighbor Gaussian Process (NNGP). Generalizing the use of nearest neighbors from expedient likelihood evaluations as in Vecchia (1988) and Stein et al. (2004) to the well-defined NNGP on the entire domain enables fully Bayesian inference and coherent recovery of the latent SREs.

Using an NNGP, the model can be written as $\varvec{Y} \sim N(\varvec{X}\varvec{\beta }, \varvec{\widetilde{\Sigma }} (\varvec{\phi }) )$ where $\varvec{\widetilde{\Sigma }}$ is the NNGP covariance matrix derived from the full GP. A Bayesian specification is completed by specifying priors for the parameters $\varvec{\beta }$ and $\varvec{\phi }$. For this application, the covariance function $\mathbb C$ consists of an stationary exponential GP with variance $\sigma ^2$ and range $\phi$ and a nugget process with variance $\sigma ^2_\varepsilon$ (see (5)). We assign a normal prior for $\varvec{\beta }$, inverse gamma priors for $\sigma ^2_w$ and $\sigma ^2_\varepsilon$ and a uniform prior for $\phi$. A Gibbs sampler for the model involves conjugate updates for $\varvec{\beta }$ and metropolis random walk updates for $\varvec{\phi }= (\sigma ^2_w, \sigma ^2_\varepsilon , \phi )'$.

Letting $\alpha = \sigma ^2_\varepsilon /\sigma ^2_w$, the model can also be expressed as $\varvec{Y} \sim N(\varvec{X}\varvec{\beta }, \sigma ^2_w \varvec{\widetilde{R} } (\phi ,\alpha ) )$ where $\varvec{\widetilde{R} }$ is the NNGP matrix derived from $\varvec{C}(\phi ) + \alpha \varvec{I}$, $\varvec{C}(\phi )$ being the correlation matrix of the exponential GP. Fixing $\alpha$ and $\phi$ gives a conjugate normal–inverse Gamma posterior distribution for $\varvec{\beta }$ and $\sigma ^2_w$. Predictive distributions for *y*(*s*) at new locations can also be obtained as *t*-distributions. The fixed values of $\alpha$ and $\phi$ can be chosen from a grid-search by minimizing root-mean-square predictive error score based on *K*-fold cross-validation. This hybrid approach departs from fully Bayesian philosophy by using hyper-parameter tuning. However, it offers a pragmatic solution for massive spatial datasets. We refer to this model as the *conjugate NNGP* model and will be the model used in this computation. Detailed algorithms for both the models are provided in Finley et al. (2018). NNGP models for analyzing massive spatial data are available on CRAN as the R-package *spNNGP* (Finley et al. 2017).

#### Periodic Embedding

When the observation locations form a regular grid, and the model is stationary, methods that make use of the discrete Fourier transform (DFT), also known as spectral methods, can be statistically and computationally beneficial, since the DFT is an approximately decorrelating transform, and it can be computed quickly and with low memory burden using fast Fourier transform (FFT) algorithms. For spatially gridded data in two or higher dimensions—as opposed to time series data in one dimension—there are two prominent issues to be addressed. The first is edge effects, and the second is missing values. By projecting onto trigonometric bases, spectral methods essentially assume that the process is periodic on the observation domain, which leads to bias in the estimates of the spectrum (Guyon 1982; Dahlhaus and Künsch 1987). Guinness and Fuentes (2017) and Guinness (2017) propose the use of small domain expansions and imputing data in a periodic fashion on the expanded lattice. Imputation-based methods also solve the second issue of missing values, since the missing observations can be imputed as well.

The methods presented here follow the iterative semiparametric approach in Guinness (2017). Guinness and Fuentes (2017) provide an alternative parametric approach. For this section, let $\varvec{N} = (N_1,N_2)$ give the dimensions of the observation grid [in the case study datasets below $\varvec{N} = (300,500)$]. Let $\tau$ denote an expansion factor, and let $m = \lfloor \tau \varvec{N} \rfloor$ denote the size of the expanded lattice. We use $\tau = 1.2$ in all examples, so that $m = (360,600)$ in the surface temperature dataset. Let $\varvec{U}$ be the vector of observations, and $\varvec{V}$ be the vector of missing values on the grid of size *m*, making the full vector $\varvec{Y} = (\varvec{U}',\varvec{V}')'$. The discrete Fourier transform of the entire vector is

$$\begin{aligned} J(\varvec{\omega }) = \frac{1}{\sqrt{m_1 m_2}} \sum _{\varvec{s}} Y(\varvec{s}) \exp (-i\varvec{\omega }'\varvec{s}), \end{aligned}$$

$\varvec{\omega } = (\omega _1,\omega _2)'$ is a spatial frequency with $\omega _j \in [0,2\pi ]$, $i = \sqrt{-1}$, and $\varvec{\omega }'\varvec{s} = \omega _1 s_1 + \omega _2 s_2$.

The procedure is iterative. At iteration *k*, the spectrum $f_k$ is updated with

15 $$\begin{aligned} f_{k+1}(\omega ) = \sum _{\varvec{\nu }} E_k( |J(\varvec{\nu })|^2 \, | \, \varvec{U} ) \alpha ( \varvec{\omega } - \varvec{\nu } ), \end{aligned}$$

where $\alpha$ is a smoothing kernel, and $E_k$ is expected value under the multivariate normal distribution with stationary covariance function

$$\begin{aligned} R_k( \varvec{h} ) = \frac{1}{m_1 m_2} \sum _{\varvec{\omega } \in \mathbb {F}_m } f_k(\varvec{\omega }) \exp (i \varvec{\omega } ' \varvec{h}), \end{aligned}$$

where $\mathbb {F}_m$ is the set of Fourier frequencies on a grid of size *m*. This is critical since it ensures that $R_k$ is periodic on the expanded grid. In practice, the expected value in (15) is replaced with $|J(\varvec{\nu })|^2$ computed using an imputed vector $\varvec{V}$, a conditional simulation of missing values given $\varvec{U}$ under covariance function $R_k$. This ensures that the imputed vector $\varvec{V}$ is periodic on the expanded lattice and reduces edge effects. The iterative procedure can also be run with an intermediate parametric step in which the Whittle likelihood (Whittle 1954) is used to estimate a parametric spectral density, which is used to filter the imputed data prior to smoothing the spectrum. See Guinness (2017) for details about more elaborate averaging schemes and monitoring for convergence of the iterative method.

### Algorithmic Approaches

#### Metakriging

Spatial metakriging is an approximate Bayesian method that is not tied to any specific model and is partly algorithmic in nature. In particular, any spatial model described above can be used to draw inference from subsets (as described below). From (1), let the $N\times N$ covariance matrix be determined by a set of covariance parameters $\varvec{\phi }$ such that ${\varvec{\Sigma }}= {\varvec{\Sigma }}(\varvec{\phi })$ (e.g., $\varvec{\phi }$ could represent decay parameters from the Matérn covariance function) and $\mu (\varvec{s}) = \varvec{X}'(\varvec{s})\varvec{\beta }$ where $\varvec{X}(\varvec{s})$ is a set of known covariates with unknown coefficients ${\varvec{\beta }}$. Further, let the sampled locations $\mathcal {S}=\{{\varvec{s}}_1, \ldots ,{\varvec{s}}_N\}$ be partitioned into sets $\{\mathcal {S}_1, \ldots ,\mathcal {S}_K\}$ such that $\mathcal {S}_i\cap \mathcal {S}_j=\emptyset$ for $i\ne j$, $\bigcup _{i=1}^K\mathcal {S}_i=\mathcal {S}$ and the corresponding partition of the data be given by $\{{\varvec{y}}_k, {\varvec{X}}_k\}$, for $k=1,2,\ldots ,K$, where each ${\varvec{y}}_k$ is $n_k\times 1$ and ${\varvec{X}}_k$ is $n_k\times p$. Assume that we are able to obtain posterior samples for ${\varvec{\Omega }}= \{{\varvec{\beta }}, {\varvec{\phi }}\}$ from (1) applied independently to each of *K* subsets of the data in *parallel on different cores*. To be specific, assume that ${\varvec{\Omega }}_k = \{{\varvec{\Omega }}_k^{(1)}, {\varvec{\Omega }}_k^{(2)},\ldots , {\varvec{\Omega }}_k^{(M)}\}$ is a collection of *M* posterior samples from $p({\varvec{\Omega }}\,|\,{\varvec{y}}_k)$. We refer to each $p({\varvec{\Omega }}\,|\,{\varvec{y}}_k)$ as a “subset posterior.” The metakriging approach we outline below attempts to combine, optimally and meaningfully, these subset posteriors to arrive at a legitimate probability density. We refer to this as the “metaposterior.”

Metakriging relies upon the unique geometric median (GM) of the subset posteriors (Minsker et al. 2014; Minsker 2015). For a positive-definite kernel $h(\cdot )$, define the norm between two distributions $\pi _1(\cdot )$ and $\pi _2(\cdot )$ of ${\varvec{\Omega }}$ by $d_{h}(\pi _1(\cdot ),\pi _2(\cdot ))=\Vert \int h({\varvec{\Omega }},\cdot )d(\pi _1-\pi _2)({\varvec{\Omega }})\Vert$. We envision the individual posterior densities $p_k \equiv p({\varvec{\Omega }}\,|\,{\varvec{y}}_k)$ to be residing on a Banach space $\mathcal{H}$ equipped with norm $d_h(\cdot ,\cdot )$. The GM is defined as

16 $$\begin{aligned} \pi ^*({\varvec{\Omega }}\,|\,{\varvec{y}}) = \arg \min \limits _{\pi \in \mathcal {H}}\sum _{k=1}^{K}d_{h}(p_k,\pi )\; , \end{aligned}$$

where ${\varvec{y}}= ({\varvec{y}}_1', {\varvec{y}}_2',\ldots ,{\varvec{y}}_K')'$. In what follows, we assume $h(z_1,z_2)=\exp (-||z_1-z_2||^2)$.

The GM is unique. Further, the geometric median lies in the convex hull of the individual posteriors, so $\pi ^*({\varvec{\Omega }}\,|\,{\varvec{y}})$ is a legitimate probability density. Specifically, $\pi ^*({\varvec{\Omega }}\,|\,{\varvec{y}})=\sum _{k=1}^{K} \xi _{h,k}({\varvec{y}})p_k$, $\sum _{k=1}^{K}\xi _{h,k}({\varvec{y}})=1$, each $\xi _{h,k}({\varvec{y}})$ being a function of $h,{\varvec{y}}$, so that $\int _{{\varvec{\Omega }}}\pi ^*({\varvec{\Omega }}\,|\,{\varvec{y}})\hbox {d}{\varvec{\Omega }}=1$.

Computation of the geometric median $\pi ^*\equiv \pi ^*({\varvec{\Omega }}\,|\,{\varvec{y}})$ proceeds by employing the popular Weiszfeld’s iterative algorithm that estimates $\xi _{h,k}({\varvec{y}})$ for every *k* from the subset posteriors $p_k$. To further elucidate, we use a well known result that the geometric median $\pi ^*$ satisfies, $\pi ^*=\left[ \sum _{k=1}^{K}p_k/d_h(p_k,\pi ^*)\right] \left[ \sum _{k=1}^{K}1/d_h(p_k,\pi ^*)\right] ^{-1}$ so that $\xi _{h,k}({\varvec{y}})= (1/d_h(p_k,\pi ^*))/ \sum _{j=1}^{K}(1/d_h(p_j,\pi ^*))$. Since there is no apparent closed form solution for $\xi _{h,k}({\varvec{y}})$ that satisfies this equation, one needs to resort to the Weiszfeld iterative algorithm outlined in Minsker et al. (2014) to produce an empirical estimate of $\xi _{h,k}({\varvec{y}})$ for all $k=1,..,K$.

Guhaniyogi and Banerjee (2018) show that, for a large sample, $\pi ^*(\cdot \,|\,{\varvec{y}})$ provides desirable approximation of the full posterior distribution in certain restrictive settings. It is, therefore, natural to approximate the posterior predictive distribution $p(y(s_0)\,|\,{\varvec{y}})$ by the subset posterior predictive distributions $p(y(s_0)\,|\,{\varvec{y}}_k)$. Let $\{y(s_0)^{(j,k)}\}_{j=1}^{M}$, $k=1,\ldots ,K$, be samples obtained from the posterior predictive distribution $p(y(s_0)|{\varvec{y}}_k)$ from the *k*-th subset posterior. Then,

$$\begin{aligned} p(y(s_0)\,|\,{\varvec{y}})\approx \sum _{k=1}^K\xi _{h,k}({\varvec{y}})p(y(s_0)\,|\,{\varvec{y}}_k)=\sum _{k=1}^K\xi _{h,k}({\varvec{y}})\int p(y(s_0)\,|\,{\varvec{\Omega }}, {\varvec{y}}_k)p({\varvec{\Omega }}\,|\,{\varvec{y}}_k)\hbox {d}{\varvec{\Omega }}\; , \end{aligned}$$

Therefore, the empirical posterior predictive distribution of the metaposterior is given by $\sum _{k=1}^{K}\sum _{j=1}^{M}\frac{\xi _{h,k}({\varvec{y}})}{M}1_{y(s_0)^{(j,k)}}$, from which the posterior predictive median and the 95% posterior predictive interval for the unobserved $y(s_0)$ are readily available.

The spatial metakriging approach has additional advantages over Minsker et al. (2014). Minsker et al. (2014) suggest computing the stochastically approximated posterior from each subset, which limits users from employing standard R packages to draw posterior samples from them. In contrast, metakriging allows subset posterior computation using popular R packages. Additionally, Minsker et al. (2014) mainly focuses on prediction and restricts its applicability only to i.i.d. settings. On the contrary, Guhaniyogi and Banerjee (2018) present comprehensive analysis on parameter estimation, residual surface interpolation and prediction for spatial Gaussian processes. Theoretical results supporting the proposed approach under restrictive assumptions have been presented in the supplementary material to Guhaniyogi and Banerjee (2018).

One important ingredient of spatial metakriging (SMK) is partitioning the dataset into subsets. For this article, we adopt a random partitioning scheme that randomly divides data into $K=30$ exhaustive and mutually exclusive subsets. The random partitioning scheme facilitates each subset to be a reasonable representative of the entire domain, so that each subset posterior acts as a “weak learner” of the full posterior. We have explored more sophisticated partitioning schemes and found similar predictive inference.

For the sake of definiteness, this article uses the stationary Gaussian process model for each subset inference which may lead to higher run time. Indeed, the version of metakriging approach presented here yields more accurate results when a stationary Gaussian process model is fitted in each subset. However, the metakriging approach lends much more scalability when any of the above models is employed in each subset. In fact, an extension to spatial metakriging, referred to as distributed spatial kriging (DISK) (Guhaniyogi et al. 2017), scales the non-stationary modified predictive process to millions of observations. Ongoing research on a more general extension of metakriging, coined as Aggregated Monte Carlo (AMC), involves scaling spatiotemporal varying coefficient models to big datasets.

#### Gapfill

The gapfill method (Gerber et al. 2018) differs from the other herein presented methods in that it is purely algorithmic, distribution-free, and, in particular, not based on Gaussian processes. Like other prediction methods popular within the satellite imaging community (see Gerber et al. 2018; Weiss et al. 2014 for reviews), the gapfill method is attractive because of its low computational workload. A key aspect of gapfill is that it is designed for parallel processing, which allows the user to exploit computing resources at different scales including large servers. Parallelization is enabled by predicting each missing value separately based on only a subset of the data.

To predict the value $Y(\varvec{s}_0)$ at location $\varvec{s}_0$ gapfill first selects a suitable subset $\varvec{A}=\{Y(\varvec{s}_i): \varvec{s}_i \in \mathcal {N}(\varvec{s}_0)\}$, where $\mathcal {N}(\varvec{s}_0)$ defines a spatial neighborhood around $\varvec{s}_0$. Finding $\varvec{A}$ is formalized with rules, which reassure that $\varvec{A}$ is small but contains enough observed values to inform the prediction. In this study, we require $\varvec{A}$ to have an extent of at least $5\times 5$ pixels and to contain at least 25 non-missing values. Subsequently, the prediction of $Y(\varvec{s}_0)$ is based on $\varvec{A}$ and relies on sorting algorithms and quantile regression. Moreover, prediction intervals are constructed using permutation arguments (see Gerber et al. 2018 for more details on the prediction and uncertainty intervals).

The gapfill method was originally designed for spatiotemporal data, in which case the neighborhood $\mathcal {N}(\varvec{s}_0)$ is defined in terms of the spatial and temporal dimensions of the data. As a consequence, the implementation of gapfill in the R package gapfill (Gerber 2017) requires multiple images to work properly. To mimic this situation, we shift the given images by one, two, and three pixels in both directions along the *x* and *y*-axes. Then the algorithm is applied to those 13 images in total (one original image and 12 images obtained through shifts of the original image).

#### Local Approximate Gaussian Processes

The local approximate Gaussian process (laGP, Gramacy and Apley 2015) addresses the big-*N* problem in GP regression by taking a so-called *transductive* approach to learning, where the fitting scheme is tailored to the prediction problem (Vapnik 1995) as opposed to the usual *inductive* approach of fitting first and predicting later conditional on the fit. A special case of laGP, based on nearest neighbors, is simple to describe. In order to predict at $\varvec{s}$, simply train a Gaussian process predictor on the nearest *m* neighbors to $\varvec{s}$; i.e., use the data subset $\mathcal {Y}_m = \{Y(\varvec{s}_i): \varvec{s}_i \in \mathcal {N}_m(\varvec{s})\}$, where $\mathcal {N}_m(\varvec{s})$ are the *m* closest observed locations to $\varvec{s}$ in terms of Euclidean distance. If the data-generating mechanism is not at odds with modeling assumptions (e.g., having a well-specified covariance structure), then one can choose *m* to be as large as possible, up to computational limitations, in order to obtain an accurate approximation. Observe that this use of nearest neighbors (NNs) for prediction is more akin to the classical statistical/machine learning variety, in contrast to their use in determining the global (inverse) covariance structure as described in Sect. 2.3.

Interestingly, NNs do not comprise an optimal data subset for prediction under the usual criteria such as mean-squared error. However, finding the best *m* of $N!/(m!(N-m)!)$ possible choices represents a combinatorially huge search. The laGP method generalizes this so-called nearest neighbor prediction algorithm (whose modern form in spatial statistical literature is described by Emery 2009) by approximating that search with a greedy heuristic. First, start with a NN set $\mathcal {Y}_{m_0}(\varvec{s})= \{Y(\varvec{s}_i): \varvec{s}_i \in \mathcal {N}_{m_0}(\varvec{s}))$ where $m_0 < m$, and then for $j=m_0+1,\dots ,m$ successively choose $\varvec{s}_{j}$ to augment $\mathcal {Y}_{m_0}$ building up a local design data set one point at a time according to one of several simple objective criteria related to mean-square prediction error. The idea is to repeat in this way until there are *m* observations in $\mathcal {Y}_m(\varvec{s})$. Gramacy and Apley’s preferred variation targets $\varvec{s}_{j}$ which maximizes the *reduction* in predictive variance at $\varvec{s}$. To recognize a similar *global* design criterion called *active learning Cohn* (Cohn 1996), they dubbed this criterion ALC. Qualitatively, these local ALC designs tend to have a cluster of neighbors and “satellite” points and have been shown to offer demonstrably better predictive properties than NN and even full-data alternatives especially when the data-generating mechanism is at odds with the modeling assumptions. The reason is that local fitting offers a way to cope with a certain degree of non-stationarity which is common in many real data settings.

ALC search iterations and GP updating considerations as designs are built up, are carefully engineered to lead to a method whose computations are of $\mathcal {O}(N^3)$ complexity (i.e., the same as the simpler NN alternative). A relatively modest local design size of $m=50$ typically works well. Moreover, calculations for each $\varvec{s}$ are statistically independent of the next, which means that they can be trivially parallelized. Through a cascade of multi-core, multi-node and GPU parallelization, Gramacy et al. (2014) and Gramacy and Haaland (2016) illustrated how *N* in the millions, in terms of both training and testing data sizes could be handled (and yield accurate predictors) with less than an hour of computing time. The laGP method has been packaged for R and is available on CRAN (Gramacy 2016). Symmetric multi-core parallelization (via OpenMP) and multi-node automations (via the built-in parallel package) work out-of-the box. GPU extensions are provided in the source code but require custom compilation.

A disadvantage to local modeling in this fashion is that a global predictive covariance is unavailable. Indeed, the statistically independent nature of calculation is what makes the procedure computationally efficient and parallelizable. In fact, the resulting global predictive surface, over a continuum of predictive $\varvec{s}$-locations, need not even be smooth. However, in most visual representations of predictive surfaces it can be difficult to distinguish between a genuinely smooth surface and what is plotted via the laGP predictive equations. Finally, it is worth noting that although laGP is applied here in a spatial modeling setting (i.e., with two input variables), it was designed for computer simulation modeling and has been shown to work well in input dimension as high as ten.

## The Competition

At the initial planning phase of this competition, we desired to compare a broad variety of approaches: from frequentist to Bayesian and from well-established to modern developments. In accordance with this plan, efforts were made to contact a variety of research groups with strong expertise in a method to analyze the datasets. After this outreach period, the research teams listed in Table 1 agreed to participate and implement their associated method.

**Table 1:** Research groups participating in the competition along with their selected method (competitor).

Group members	Method
Abhirup Datta and Andrew Finley	Nearest Neighbor Processes
Andrew Finley	Predictive Processes
Reinhard Furrer	Covariance Tapering
Florian Gerber	Gapfill
Raj Guhaniyogi	Metakriging
Matthew J. Heaton	Spatial Partitioning
Andrew Zammit-Mangion	Fixed Rank Kriging
Matthias Katzfuss and Dorit Hammerling	Multiresolution Approximations
Finn Lindgren	Stochastic Partial Differential Equations
Joseph Guinness	Periodic Embedding
Douglas Nychka	Lattice Kriging
Robert Gramacy and Furong Sun	Local Approximate Gaussian Processes

Each group listed in Table 1 was provided with two training datasets: one real and one simulated. The simulated dataset represented a case where the covariance function was specified correctly while the real dataset represented a scenario where the covariance function was mis-specified. Both datasets consisted of observations on the same 500$\times$300 grid ranging longitude values of $-\,95.91153$ to $-\,91.28381$ and latitude values of 34.29519 to 37.06811. The real dataset consisted of daytime land surface temperatures as measured by the Terra instrument onboard the MODIS satellite on August 4, 2016 (Level-3 data). The data were downloaded from the MODIS reprojection tool web interface (MRTweb). While this exact tool was discontinued soon after this project began, the data are provided on GitHub at https://github.com/finnlindgren/heatoncomparison. The latitude and longitude ranges, as well as the date, were chosen because of the sparse cloud cover over the region on this date (rather than by scientific interest in the date itself). Namely, only 1.1% of the Level-3 MODIS data were corrupted by cloud cover leaving 148,309/150,000 observed values to use for our purposes.

**Fig. 1:**
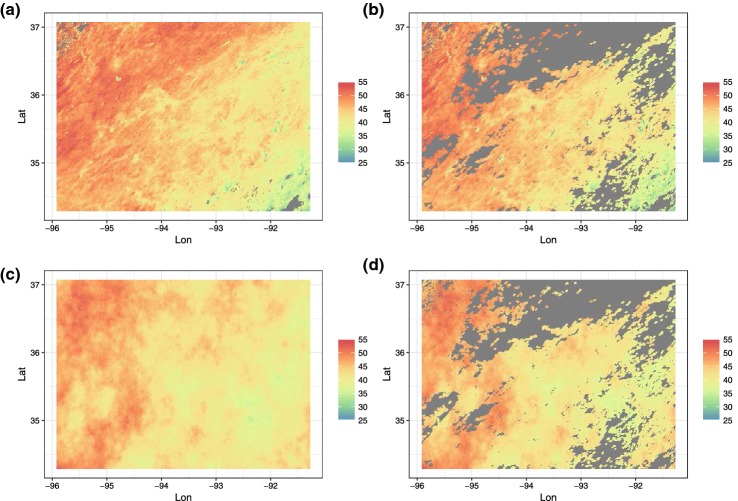
The top row displays the **a** full and **b** training satellite datasets. The bottom row displays the **c** full and **d** training simulated data.

The simulated dataset was created by, first, fitting a Gaussian process model with constant mean, exponential covariance function and a nugget effect to a random sample of 2500 observations from the above MODIS data. The resulting parameter estimates were 4 / 3, 16.40, 0.05, and 44.49 for the spatial range, spatial variance, nugget variance and constant mean, respectively. The spatial range parameter of 4 / 3 equated to an approximate effective spatial range (the distance at which the correlation is equal to 0.05) of approximately 210 miles (338 km). These parameters were then used to simulate 150,000 observations on the same grid as the MODIS data.

To define test and training sets, the missing data pattern on August 6, 2016 from the same MODIS satellite data product was used to separate each dataset into training and test sets. After the split, the training set for the MODIS data consisted of 105,569 observations leaving 42,740 observations in the test set. The training set for the simulated data also consisted of 105,569 observations but a test set size of 44,431 (the difference in test set size is contributed to missing data due to cloud cover in the original MODIS data). Research teams were provided with the training set and the locations of the test set (but not the actual observation in the test set). Figure 1 displays the full datasets along with the corresponding training set provided to each research group. All datasets used in this article are provided on the public GitHub repository https://github.com/finnlindgren/heatoncomparison.

Each group independently wrote code (also included on the accompanying GitHub page) that provided (i) a point prediction for each location in the test set, (ii) a 95% prediction interval for location in the test set or a corresponding standard error for the prediction, and (iii) the total clock time needed to implement the method. In order to minimize the number of confounding factors in this competition, each group was instructed to use an exponential correlation function (if applicable to their chosen method) and a nugget variance. For the simulated data the groups were instructed to only use a constant mean (because this was how the data were originally simulated). However, for the satellite data, the groups used a linear effect for latitude and longitude so that the residual process more closely resembled the exponential correlation. The code from each team was then run on the Becker computing environment (256 GB of RAM and 2 Intel Xeon E5-2680 v4 2.40GHz CPUs with 14 cores each and 2 threads per core - totaling 56 possible threads for use in parallel computing) located at Brigham Young University (BYU). Each team’s code was run individually and no other processes were simultaneously run so as to provide an accurate measure of computing time.

Each method was compared in terms of mean absolute error (MAE), root-mean-squared error (RMSE), continuous rank probability score (CRPS; see Gneiting and Raftery 2007; Gneiting and Katzfuss 2014), interval score (INT; see Gneiting and Raftery 2007) and prediction interval coverage (CVG; the percent of intervals containing the true value). To calculate the CRPS, we assumed the associated predictive distribution was well approximated by a Gaussian distribution with mean centered at the predicted value and standard deviation equal to the predictive standard error. In cases where only a prediction interval was provided, the predictive standard error was taken as $(U-L)/(2\times \Phi ^{-1}(0.975))$ where *U* and *L* are the upper and lower ends of the interval, respectively.

## Competition Results

### Results for Simulated Data

The numerical results for the simulated data competition are displayed in Table 2. First, consider the predictive accuracy as measured by the MAE and RMSE in Table 2. In terms of predictive accuracy, the best MAE was 0.61 while the worst was only 1.03 (68% difference). Similarly, the best RMSE was 0.83 compared to a worst RMSE of only 1.31 (a 57% difference). Yet, notably, with only a single simulated dataset these results are suggestive but not conclusive regarding which methods give consistently better predictions.

Considering uncertainty quantification (UQ) some of the methods fared better than others. For example, LatticeKrig, LAGP, metakriging, MRA, periodic embedding, NNGP, and PP all achieved near the nominal 95% coverage rate. In contrast, FRK, Gapfill, and partitioning achieved lower than nominal coverage while SPDE and tapering have higher than nominal coverage. Considering UQ further, Gapfill has a large interval score suggesting possible wide predictive intervals in addition to the penalty incurred from missing the true value. In this regard, it is important to keep in mind that LAGP, metakriging, MRA, NNGP and PP all can specify the “correct” exponential correlation function. Additionally, LK and SPDE have settings that can approximate the exponential correlation function well. In contrast, some methods such as FRK and Gapfill are less suited to model fields with exponential correlation functions, which may partially explain their relatively poor prediction or coverage performance in this instance.

**Table 2:** Numerical scoring for each competing method on the simulated data.

Method	MAE	RMSE	CRPS	INT	CVG	Run time (min)	Cores used
FRK	1.03	1.31	0.74	8.35	0.84	2.18	1
Gapfill	0.73	1.00	0.64	18.01	0.44	0.63	40
LatticeKrig	0.63	0.87	0.45	4.04	0.97	25.58	1
LAGP	0.79	1.11	0.57	5.71	0.90	2.28	40
Metakriging	0.74	0.97	0.53	4.69	0.99	2888.89	30
MRA	0.61	0.83	0.43	3.64	0.93	13.57	1
NNGP	0.65	0.88	0.46	3.79	0.96	1.99	10
Partition	0.64	0.86	0.47	5.05	0.86	77.56	55
Pred. Proc.	1.06	1.43	0.76	7.33	0.89	161.66	10
SPDE	0.62	0.86	0.59	7.81	1.00	138.34	2
Tapering	0.69	0.97	0.55	6.39	1.00	188.36	1
Periodic Embedding	0.65	0.91	0.47	4.16	0.97	13.31	1

**Fig. 2:**
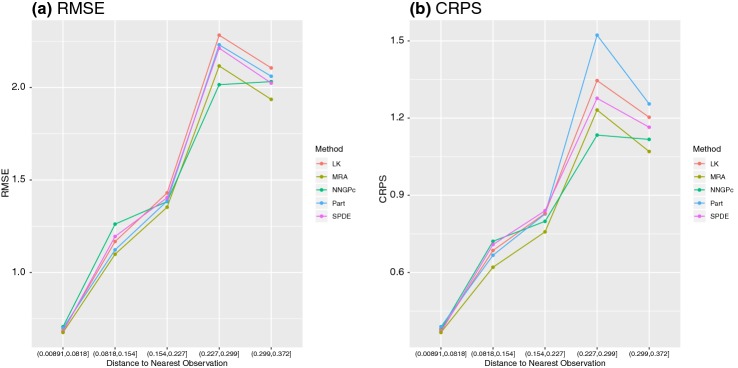
**a** RMSE and **b** CRPS by distance to the nearest observation for the top performers on the simulated dataset.

To explore differences among the methods further, we calculated RMSE and CRPS for predictions in 5 categories where the categories were created from distance to the nearest training point. The number of observations per class was 36106, 5419, 1918, 729 and 259 from shortest to longest distance categories, respectively (i.e., there were 36,106 predictions classified as “short distance”). Figure 2 displays the RMSE and CRPS of the top 5 performing methods (in terms of overall RMSE) for each prediction distance class. While there is little difference among the methods for short distance predictions, there is more spread in the methods at longer distances. That is MRA , SPDE and NNGP seem to be preferred for longest distance predictions over spatial partitioning and LK. The difference between these methods is larger when considering uncertainty (CRPS) rather than just predictive accuracy (RMSE).

### Results for Real Data

The results for the real MODIS data are displayed in Table 3 and largely reiterate the results from the simulated data. Namely, each method performed very well in terms of predictive accuracy. The largest RMSE was only 2.52 which, when considered on the data range of $55.41-24.37=31.04$, is very small. Relative to the simulated data, the observed RMSEs were considerably higher for all methods which attributed to model misspecification. We note that, under the setup of the competition, some of the methods were forced to approximate a GP with isotropic exponential covariance function, which is the true covariance function of the simulated data, but most certainly not for the real data. Thus, the scores are lowest for those approximations that happened to result in a good fit to the data and not necessarily lowest for those methods that best approximated the exponential covariance. This might also explain why MRA performed well for long-distance predictions in the simulated example but did not perform as well for long-distance satellite prediction. Further, because many of the top performing methods strive to approximate an exponential covariance, the subtle differences between the top performing methods on simulated versus real data should not be attributed to robustness in model misspecification.

**Table 3:** Numerical scoring for each competing method on the satellite data.

Method	MAE	RMSE	CRPS	INT	CVG	Run time (min)	Cores used
FRK	1.96	2.44	1.44	14.08	0.79	2.32	1
Gapfill	1.33	1.86	1.17	34.78	0.36	1.39	40
LatticeKrig	1.22	1.68	0.87	7.55	0.96	27.92	1
LAGP	1.65	2.08	1.17	10.81	0.83	2.27	40
Metakriging	2.08	2.50	1.44	10.77	0.89	2888.52	30
MRA	1.33	1.85	0.94	8.00	0.92	15.61	1
NNGP	1.21	1.64	0.85	7.57	0.95	2.06	10
Partition	1.41	1.80	1.02	10.49	0.86	79.98	55
Pred. Proc.	2.15	2.64	1.55	15.51	0.83	160.24	10
SPDE	1.10	1.53	0.83	8.85	0.97	120.33	2
Tapering	1.87	2.45	1.32	10.31	0.93	133.26	1
Periodic Embedding	1.29	1.79	0.91	7.44	0.93	9.81	1

The largest discrepancies among the competing methods is again in terms of uncertainty quantification. Lattice kriging, metakriging, MRA, NNGP and periodic embedding again achieved near nominal coverage rates with small interval scores and CRPS. The SPDE and tapering approaches did better in terms of coverage in that the empirical rates were near nominal (recall that the corresponding coverage rates were too high for the simulated data for these methods). In contrast, the coverage rates on the MODIS data for FRK, Gapfill, LAGP, partitioning and PP were too small resulting in larger interval scores.

**Fig. 3:**
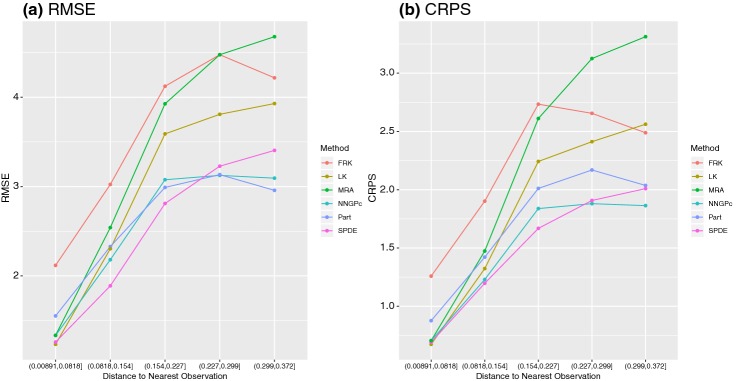
**a** RMSE and **b** CRPS by distance to the nearest observation for the top performers on the satellite dataset.

Figure 3 displays the results for RMSE and CRPS as a function of distance category for the 5 top performing methods (in terms of overall RMSE) and one low-rank method (FRK) in the satellite case study. When considering prediction distance, more noticeable differences are found between the methods in this real data application as opposed to the simulated data application. NNGP and SPDE perform consistently well across all distance categories for both the simulated and satellite data. Further, it is apparent from this plot that prediction performance of low-rank methods is inferior (see Table 3) because they do not perform well for short-range predictions (this was expected for FRK, where the number of basis functions used is relatively small). However, they still do well, comparatively, when predicting over large gaps.

## Conclusions

The contribution of this article was fourfold: (i) provide an overview of the plethora of methods available for analyzing large spatial datasets, (ii) provide a brief comparison of the methods by implementing a case study competition among research groups, (iii) make available the code to analyze the data to the broader scientific community and (iv) provide an example of the common task framework for future studies to follow when comparing various analytical methods. In terms of comparison, each of the methods performed very well in terms in predictive accuracy suggesting that any of the above methods are well suited to the task of prediction. However, the methods differed in terms of their ability to accurately quantify the uncertainty associated with the predictions. While we saw that some methods did consistently well in both predictive performance and nominal coverage on the simulated and real data, in general we can expect performance of any method to change with size of the dataset, measurement-error variance, and the nature of missingness. Further, while the results in Table 1 are suggestive, with only one simulated and one real dataset we cannot definitively claim that any one method provides consistently better predictions than any other method. However, the data scenario’s considered here are relatively representative of a typical spatial analysis such that our results can be used as a guide for practitioners.

Each of the above methods performed well for both scenarios considered in this paper. However, situations where each respective method does not perform well are also of interest. For example, it is known that low-rank methods such as FRK and predictive processes will struggle with high signal-to-noise ratio and where the process has a small spatial range (as was seen here for the simulated data; see Zammit-Mangion and Cressie 2018; Zammit-Mangion et al. 2018). The gapfill method may struggle if the data are not on a regular grid. Moreover, depending on the parameters and the pattern of missing values in the data the predictions from gapfill, LAGP and spatial partitioning may show discontinuities. Likewise, it is know that metakriging approach described here is less accurate when each subset uses a non-stationary GP instead of a stationary GP but recent research seeks to remedy this issue (Guhaniyogi et al. 2017).

At the outset of this study, run time and computation time for each method was of interest. However, because many of these methods are very young in their use and implementation, the variability across run time was too great to be used as a measure to compare the methods. For example, some methods are implemented in R while others are implemented in MATLAB. Still, others use R as a front end to call C-optimized functions. Hence, while we reported the run times in the results section, we provide these as more of an “off the shelf” run time estimate rather than an optimized run time. Until time allows for each method to be further developed and software becomes available comparing run times can be misleading.

Importantly, no effort was made to standardize the time spent on this project by each group. Some groups were able to quickly code up their analysis from existing R or MATLAB libraries. Others, however, had to spend more time writing code specific to this analysis. Undoubtedly, some groups likely spent more time running “in house” cross-validation studies to validate their model predictions prior to the final run on the BYU servers while others did not. Because of this difference, we note that some of the discrepancies in results seen here may be attributable to the amount of effort expended by each group. However, we still feel that the results displayed herein give valuable insight into the strengths and weaknesses of each method.

This study, while thorough, is non-comprehensive in that other methods for large spatial data (e.g., Sang and Huang 2012; Stein et al. 2013; Kleiber and Nychka 2015; Castrillon-Candás et al. 2016; Sun and Stein 2016; Litvinenko et al. 2017) were not included. Additionally, methods are sure to be developed in the future which are also viable for modeling large spatial data (see Ton et al. 2017; Taylor-Rodriguez et al. 2018). We made attempts to invite as many groups as possible to participate in this case study but, due to time and other constraining factors, not all groups were able to participate. However, in our opinion, the methods compared herein are representative of the most common methods for large spatial data at the time of writing.

We note that the data scenarios considered in this case study do not cover the spectrum of issues related to spatial data. That is, spatial data may exhibit anisotropy, non-stationarity, large and small range spatial dependence as well as various signal-to-noise ratios. Hence, we note that further practical distinctions between these various methods could be made depending on their applicability to these various spatial data scenarios. However, the comparison included here serves as a nice baseline case for method performance. Further research can develop case study competitions for these more complicated scenarios.

Notably, each method was compared only in terms of predictive accuracy. Further comparisons could include estimation of underlying model parameters. The difficulty in comparing estimation, however, is that not all the methods use the same model structure. For example, NNGP uses an exponential covariance while Gapfill does not require a specified covariance structure. Hence, we leave the comparison of the parameter estimates to a future study.

This comparison focused solely on spatial data. Hence, we stress that the results found here are applicable only to the spatial setting. However, spatiotemporal data are often considerably larger and more complex than spatial data. Many of the above methods have extensions to the space time setting (e.g., Gapfill is built directly for spatiotemporal settings). Further research is needed to compare these methods in the spatiotemporal setting.

## Electronic supplementary material

Below is the link to the electronic supplementary material.

**Supplementary Materials ** Additional details regarding the implementation of some of the methods to the training datasets.
