# Supplementary material for: A Case Study Competition Among Methods for Analyzing Large Spatial Data
Source: J Agric Biol Environ Stat. 2018 Dec 14;24(3):398–425. doi: 10.1007/s13253-018-00348-w (PMC6709111; doi:10.1007/s13253-018-00348-w)
Supplement: Supplementary file 1 — Supplementary Materials Additional details regarding the implementation of some of the methods to the training datasets. [file 13253_2018_348_MOESM1_ESM.pdf]

Computational Detail

April 2, 2018

1. ADDITIONAL IMPLEMENTATION DETAILS

1.1 Fixed Rank Kriging

In order to cater easily for datasets of differing support, FRK discretizes the spatial domain $\mathcal{D}$ into M small basic areal units (BAUs, e.g., Nguyen et al. 2012) where M is large, typically much greater than N. Every data point is 'binned' into one of these BAUs such that the process $Y(\cdot)$ is discretized on this grid into the vector $\boldsymbol{Y} = (Y_1, \ldots, Y_M)'$. A similar discretization of $\boldsymbol{X}(\cdot)$, $w(\cdot)$ and $\varepsilon(\cdot)$ on the grid yields the finite-dimensional system

$$\boldsymbol{Y} = \boldsymbol{X}\boldsymbol{\beta} + \boldsymbol{w} + \boldsymbol{\varepsilon},$$
$$\boldsymbol{Z} = \boldsymbol{C}\boldsymbol{Y} + \boldsymbol{\xi},$$

where $\boldsymbol{C}$ is an incidence matrix that maps the process $\boldsymbol{Y}$ to $\boldsymbol{Z}$, $\boldsymbol{\varepsilon}$ is white noise with diagonal variance $\sigma_\varepsilon^2\boldsymbol{V}$ where the diagonal elements $\boldsymbol{V}_\xi$ are specified by the user and $\boldsymbol{\xi}$ is measurement error with variance $\sigma_\xi^2\boldsymbol{I}$.

In FRK, the small-scale effect $\boldsymbol{w} = \boldsymbol{H}\boldsymbol{\theta}$, where $\boldsymbol{H} = \{h_k(\boldsymbol{s}_i)\}_{i,k}$ is the $M \times (\sum_{r=1}^{R} K_r)$ matrix of spatial basis functions (K_r basis functions at the rth resolution) and $\mathbb{V}\text{ar}(\boldsymbol{\theta}) = \boldsymbol{S}(\boldsymbol{\phi})$ with covariance parameters $\boldsymbol{\phi}$ that need to be estimated. By default in FRK, $\boldsymbol{S}(\boldsymbol{\phi})$ is a block-diagonal matrix

of R matrices where the rth block has i, jth element $\exp(-d_r(i,j)/\phi_r)$ and $d_r(i,j)$ is the distance between the centroids of the ith and j^{th} basis function at the rth resolution, and $\boldsymbol{\phi} = (\phi_1, \ldots, \phi_R)'$ are the spatial correlation parameters of the exponential correlation function. Alternatively, $\boldsymbol{S}(\boldsymbol{\phi})$ can be unstructured in which case $K(K+1)/2$ parameters need to be estimated.

Parameter estimation of $\{\sigma^2_\varepsilon, \sigma^2_\xi, \boldsymbol{\beta}, \boldsymbol{\theta}, \boldsymbol{\phi}\}$ in `FRK` proceeds as follows. First, since the measurement error variance and fine-scale variance are confounded, σ^2_ξ is initially estimated from the data using variogram techniques as described in Kang et al. (2009). Second, an expectation maximization algorithm is used, which infers $\boldsymbol{\theta}$ in the E-step, and estimates $\{\sigma^2_\varepsilon, \boldsymbol{\beta}, \boldsymbol{\phi}\}$ in the M-step. Following estimation, prediction proceeds by finding the distribution of $\boldsymbol{Y} \mid \boldsymbol{Z}$. Computations are facilitated throughout via the Sherman–Morrison–Woodbury matrix identity and a matrix-determinant lemma.

In `FRK` the user may specify several options, such as the number of basis functions, the basis-function type, the specific BAUs to use, etc. In this competition, the data was supplied as an `R` data frame containing grid points. These grid points can be used to construct a `SpatialPixelsDataFrame` object which can then be used as BAUs. The diagonal elements of $\boldsymbol{V}_\varepsilon$ are assigned to the BAUs using the field `fs`. Since in this example we do not have any prior knowledge as to what could affect the fine-scale variation, we set this field to one.

```
library(sp)
BAUs <- sat.temps             # set as BAUs
BAUs$Temp <- NULL          # remove temperature data from BAUs
coordinates(BAUs)  <- ~Lon+Lat   # make SpatialPointsDataFrame
gridded(BAUs) <- TRUE    # make SpatialPixelsDataFrame
```

The data supplied to `FRK` must not contain any missing values and must be a `Spatial` object, in this case a `SpatialPointsDataFrame`:

```
dat <- subset(sat.temps,!is.na(Temp))  # remove missing data
```

```
coordinates(dat)  <- ~Lon+Lat     # make SpatialPointsDataFrame
```

The basis functions are constructed using the function `auto_basis`. This function is supplied with the manifold (in this case `plane()`), the data, the number of basis-function resolutions (`nres=3`) and a flag indicating that we wish to have the basis functions irregularly spaced in the domain (`regular=0`). By default, the basis functions are bisquare functions.

FRK is run using the function `FRK`. It is supplied with a formula `f` relating the response variable to covariates (the intercept is included by default), the data, the basis functions, and the BAUs, There are several other options one can use, see `help(FRK)` for details. The function `FRK` returns an object of class `SRE`.

```
S <- FRK(f = Temp ~ Lon + Lat,  # R formula
         data = dat,            # data
         BAUs = BAUs,           # BAUs
         basis = basis,         # 3 resolutions
         regular = 0)           # irregular basis functions
```

Prediction on all BAUs (which includes all missing-data locations) proceeds using the function `SRE.predict` on the SRE object. In this case the returned BAUs are of class `SpatialPixelsDataFrame` which can be converted to a normal data frame using the `data.frame` command. The resulting data frame has both the coordinate locations as well as the prediction mean, prediction error, and prediction variance.

```
BAUs_pred <- SRE.predict(S)                # predict over all BAUs
BAUs_pred_df <- data.frame(BAUs_pred)    # convert to data frame
```

1.2 Lattice Kriging

The rectangular grid of node points $\{\boldsymbol{u}_{rk}\}$ are described by the `LatticeKrig` parameters `NC`, `overlap`, `nbuffer`,and `nlevel`. `NC` specifies the number of grid points in the spatial

domain for the coarsest level and along the longest dimension. `overlap` is the scaling of the basis functions relative to the node spacing with a default value of 2.5. `nbuffer` is the number of extra grid points, also equally spaced, added at the four margins and at each level (default is 5 for all levels). Finally, `nlevel` is the number of multi-resolution levels. Since these are the variables used in the `LatticeKrig` R package we will use R formatted equations to show their relationships.

To simplify assume the spatial domain is square. The grid at the coarsest level will be `(NC + 2*nbuffer)*(NC + 2*nbuffer)` node points For the domain $[0,1] \times [0,1]$ the spacing of node points will be `1/( NC -1)` and in general we refer to the spacing of the nodes at the coarsest level by the variable `delta`.

The next level will subdivide the `delta` spacing by a factor of 2 and result in `2*NC -1` node points along each dimension with again `2*nbuffer` points added on each margin. Subsequent grids are defined by spacings `delta[r] = delta*2^( -r+1 )` and yield a sequence of grids $\{\boldsymbol{u}_{rk}\}$ that increase roughly by a factor of four in size from level r to level $r+1$.

To define the basis functions for the rth level we take

$$\theta_r = \texttt{overlap*delta[r]}$$

and define the radial basis functions as in Equation (2.5) of the paper.

The SAR model for $\boldsymbol{Q}_r^{-1}$ is defined as follows. Let $\boldsymbol{B}$ be the SAR matrix that is square with the same dimension as $\boldsymbol{\theta}_r$. The diagonal elements of $\boldsymbol{B}$ are parametrized by $4 + 1/\phi_r^2$. In the `LatticeKrig` R package the parameter `a.wght` is defined as this diagonal value and is used instead of ϕ_r). For the kth row of $\boldsymbol{B}$ the entries corresponding to the four nearest neighbors of $\boldsymbol{u}_{rk}$ are set to -1 and the remaining elements are set to zero. With this matrix set $\boldsymbol{Q}_r = \boldsymbol{B}^T\boldsymbol{B}$ and we assume that $\boldsymbol{\theta}_r$ is distributed multivariate normal with mean $\boldsymbol{0}$ and covariance matrix $\boldsymbol{Q}_r^{-1}$.

The `LatticeKrig` model has a varied set of parameters and determining values for these

based on the data were handled in several stages. Given the covariance and basis parameters `NC`, `nlevel`, `nu` and `a.wght`, the variance parameters, σ_ε^2 and σ_w^2 and the fixed effects $\boldsymbol{\beta}$ are found by maximum likelihood. The choice of `NC`, `nlevel`, `nu` and `a.wght` was based on the criteria:

- Choosing `NC` and `nlevel` so that the number of basis functions is comparable to the number of spatial observations.
- Refining the subset of `NC`, `nlevel`, `nu` and `a.wght` values based on least squares fitting of the model variogram to the empirical variogram of the data.
- Using cross-validation to evaluate the out-of-sample prediction error for each model. To do so, two kinds of cross-validation were entertained: (i) omitting a random sample of 10% of the training data set and predicting these based on the remaining 90% and (ii) omitting a larger contiguous patch of data comparable to the missingness pattern in the actual data set and predicting these omitted data points.

Based on the analysis the models used for prediction were: `NC=30, nlevel=4,nu=.1, a.wght=4.4` for the simulated data and `NC=40, nlevel=4, nu=.1, a.wght=10.25` for the satellite data.

1.3 Local Approximate Gaussian Processes

One challenge to deploying `laGP` in the setting of this competition is that the training data are not uniformly spaced relative to the testing locations (see Figure 2 of the main article), and therefore a predictive location $\boldsymbol{s}$ could be far from its nearest neighbors. To address this, the `laGP` code used for this study trained the local predictor not on the raw data but on residuals from a global GP fit. Of course, the large N thwarts a full GP being deployed in this manner, and in any case using a full GP may not be ideal for other reasons. We found it most useful to focus the "global GP" on the task of capturing large-scale effects when the goal is extrapolating training-data-poor parts of the testing space. Therefore we trained the global GP with a $n = 100$-sized

maximum entropy sub-design (Chaloner and Verdinelli 1995) from the training set. In addition to defining the residuals on which the `laGP` is trained, we used the estimated (square-root of) spatial range parameters from the "global GP" to pre-scale the input space so that `laGP`s on the residuals could be initialized with a range of `1`. Subsequently, the `laGP`s were allowed to adapt to their local designs in the usual way. The resulting predictor, combining global and local ranges with local fitting on residuals, thereby takes on a multi-resolution effect that has been shown to yield highly accurate predictors—better than ordinary `laGP` ones—in many settings (Gramacy 2016, Section 3.2). Predictions from the global/local hybrid is facilitated by adding their respective point predictions and combining the variances for the mean, in the case of the global model, and their full variance from the `laGP` fits.

1.4 Gap Filling

The gapfill method is implemented in the programming languages R/C++ and is available as open-source R package `gapfill` (Gerber 2017). Since the method predicts each missing value separately by taking only a subset of the data into account, it is straightforward to parallelize the algorithm. We use tools from the R package `foreach` (Analytics and Weston 2015) to parallelize the prediction via OpenMP (OpenMP architecture review board 2016) or MPI (MPI Forum 2016) back-ends. More information about the usage and implementation of `gapfill` is given in the reference manual of the R package. The manual also highlights the flexible software design, which allows the user to easily modify or replace large parts of the algorithm in order to optimize it for specific datasets.

The following R code was used to predict the missing values of the satellite dataset:

```
library("gapfill")
library("doParallel")
registerDoParallel(40) # run 40 tasks in parallel
library("abind")

## load data
```

```
load("data/SatelliteTemps.RData")

## rearrange data as matrix
data <- with(sat.temps,
             array(Temp,
                   c(length(unique(Lon)), length(unique(Lat)))))
dim <- dim(data)
nx <- dim[1]; ny <- dim[2]

## display data
## Image(data)

## augment data: since the gapfill method is designed for
## spatio-temporal data, we artificially create 9 additional
## and similar images by shifting the given image
data_augmented <- abind(data,
                        data[c(1,1:(nx-1)),],
                        data[,c(1,1:(ny-1))],
                        data[c(2:nx,nx),],
                        data[,c(2:ny,ny)],

                        data[c(2:nx,nx),c(2:ny,ny)],
                        data[c(2:nx,nx),c(1,1:(ny-1))],
                        data[c(1,1:(nx-1)),c(2:ny,ny)],
                        data[c(1,1:(nx-1)),c(1,1:(ny-1))],

                        data[c(3:nx,nx,nx),],
                        data[,c(3:ny,ny,ny)],
                        data[c(1,1,1:(nx-2)),],
                        data[,c(1,1,1:(ny-2))],
                        along = 3)
dim(data_augmented) <- c(nx, ny, dim(data_augmented)[3], 1)

## predict missing values
out <- Gapfill(data_augmented,
               ## only predict missing values in first image
               subset = which(is.na(data_augmented[,,1,1])),
               ## use parallel processing via R package foreach
               dopar = TRUE,
               ## tuning parameters of the algorithm
               initialSize = c(2L, 2L, 100L, 100L),
```

```
                nTargetImage = 2,
                nQuant = 3,
                ## restrict values to the following range
                clipRange = range(data_augmented[,,1,1], na.rm=TRUE),
                ## return prediction interval
                nPredict = 3, predictionInterval = TRUE)

## extract prediction and prediction interval
prediction <- out$fill[,,1,1,1]
ciLo <- out$fill[,,1,1,2]
ciUp <- out$fill[,,1,1,3]
```

REFERENCES

Analytics, R. and Weston, S. (2015), *foreach: Provides Foreach Looping Construct for R*, R package version 1.4.3.

Chaloner, K. and Verdinelli, I. (1995), "Bayesian Experimental Design: A Review," *Statistical Science*, 10, 273–304.

Gerber, F. (2017), *gapfill: Fill Missing Values in Satellite Data*, r package version 0.9.5.

Gramacy, R. B. (2016), "laGP: Large-Scale Spatial Modeling via Local Approximate Gaussian Processes in R," *Journal of Statistical Software*, 72, 1–46.

Kang, E., Liu, D., and Cressie, N. (2009), "Statistical analysis of small-area data based on independence, spatial, non-hierarchical, and hierarchical models," *Computational Statistics & Data Analysis*, 53, 3016–3032.

MPI Forum (2016), "Message Passing Interface (MPI) forum," .

Nguyen, H., Cressie, N., and Braverman, A. (2012), "Spatial statistical data fusion for remote sensing applications," *Journal of the American Statistical Association*, 107, 1004–1018.

OpenMP architecture review board (2016), “OpenMP application program interface, version 4.5,”
.
